# Supplementary material for: Associations between historical redlining and birth outcomes from 2006 through 2015 in California
Source: PLoS One. 2020 Aug 7;15(8):e0237241. doi: 10.1371/journal.pone.0237241 (PMC7413562; doi:10.1371/journal.pone.0237241)
Supplement: S1 Table — Abbreviations: MHV-median home value; %HS Educated-percentage of residents who completed high school, some college, or all of college; %-percent. All correlation coefficients were statistically significant (p<0.05). (DOCX) [file pone.0237241.s001.docx]

**S1 Table. Spearman’s correlation coefficient matrix of 1940s areal apportioned covariates across all births.**

|  | MHV | % Non-White | % Major repairs | % Employed | % Radio Ownership | Persons per unit | % Refrigerator Ownership | %HS Educated | % Black | % Foreign-born white |
| --- | --- | --- | --- | --- | --- | --- | --- | --- | --- | --- |
| MHV |  | -0.21 | 0.04 | 0.04 | -0.06 | 0.86 | -0.31 | -0.33 | -0.36 | -0.21 |
| % Non-White |  |  | 0.22 | -0.09 | -0.22 | -0.10 | 0.60 | 0.64 | 0.22 | 0.94 |
| % Major repairs |  |  |  | -0.33 | -0.24 | 0.04 | 0.10 | 0.12 | 0.20 | 0.18 |
| % Employed |  |  |  |  | -0.22 | 0.07 | -0.04 | -0.07 | -0.01 | -0.07 |
| % Radio Ownership |  |  |  |  |  | -0.09 | -0.17 | -0.27 | -0.10 | -0.20 |
| Persons-per-unit |  |  |  |  |  |  | -0.21 | -0.23 | 0.38 | -0.12 |
| % Refrigerator Ownership |  |  |  |  |  |  |  | 0.73 | -0.10 | 0.66 |
| % with HS Education |  |  |  |  |  |  |  |  | -0.11 | 0.66 |
| % Black |  |  |  |  |  |  |  |  |  | 0.05 |
| % Foreign-born white |  |  |  |  |  |  |  |  |  |  |

Abbreviations: MHV-median home value; %HS Educated-percentage of residents who completed high school, some college, or all of college; %-percent. All correlation coefficients were statistically significant (p<0.05).
