# Supplementary material for: Associations between historical redlining and birth outcomes from 2006 through 2015 in California
Source: PLoS One. 2020 Aug 7;15(8):e0237241. doi: 10.1371/journal.pone.0237241 (PMC7413562; doi:10.1371/journal.pone.0237241)
Supplement: S2 Table — Model 1 was adjusted for MHV, % employed, %non-white, and % with radio ownership; Model 2 was additionally adjusted for maternal age, parity, and birth year. (DOCX) [file pone.0237241.s002.docx]

**S2 Table. Propensity score matched analysis odds ratio sensitivity analysis.**

|  | B vs. A | C vs. B | D vs. C |  |
| --- | --- | --- | --- | --- |
| Model 1 | 1.02 (0.94, 1.10) | 1.02 (1.00, 1.05) | 0.93 (0.91, 0.95) | PTB |
| Model 2 | 1.02 (0.94, 1.10) | 1.01 (0.99, 1.04) | 0.93 (0.91, 0.95) |  |
| Model 1 | 0.90 (0.82, 0.98) | 1.02 (0.99, 1.06) | 0.94 (0.92, 0.97) | LBW |
| Model 2 | 0.92 (0.84, 1.00) | 1.04 (1.00, 1.07) | 0.94 (0.92, 0.97) |  |
| Model 1 | 0.94 (0.88, 1.01) | 1.03 (1.00, 1.05) | 0.94 (0.92, 0.96) | SGA |
| Model 2 | 0.94 (0.88, 1.01) | 1.02 (1.00, 1.05) | 0.94 (0.92, 0.96) |  |
| Model 1 | 0.69 (0.41, 1.16) | 1.13 (0.92, 1.40) | 1.08 (0.91, 1.28) | PM |
| Model 2 | 0.70 (0.42, 1.18) | 1.12 (0.91, 1.39) | 1.10 (0.93, 1.30) |  |

Model 1 was adjusted for MHV, % employed, %non-white, and % with radio ownership;

Model 2 was additionally adjusted for maternal age, parity, and birth year
