# Supplementary material for: Associations between historical redlining and birth outcomes from 2006 through 2015 in California
Source: PLoS One. 2020 Aug 7;15(8):e0237241. doi: 10.1371/journal.pone.0237241 (PMC7413562; doi:10.1371/journal.pone.0237241)
Supplement: S3 Table — Abbreviations: VPT-very preterm; VLBW-very low birth weight; NND-neonatal death; OR-odds ratio; CI-confidence interval. Odds ratios were derived from propensity score matched analysis. (DOCX) [file pone.0237241.s003.docx]

**S3 Table. Propensity score matched analysis including very preterm (<32 weeks), very low birth weight (<1500 grams), and neonatal death.**

|  |  |  | B vs. A |  | C vs. B |  | D vs. C |
| --- | --- | --- | --- | --- | --- | --- | --- |
|  |  |  | OR (95% CI) |  | OR (95% CI) |  | OR (95% CI) |
| All births | VPT |  | 0.97 (0.79, 1.19) |  | 1.11 (1.03, 1.19) |  | 0.92 (0.87, 0.98) |
|  | VLBW |  | 0.97 (0.77, 1.20) |  | 1.08 (1.00, 1.16) |  | 0.96 (0.90, 1.02) |
|  | NND |  | 0.74 (0.33, 1.69) |  | 1.45 (1.03, 2.04) |  | 1.13 (0.88, 1.45) |

Abbreviations: VPT-very preterm; VLBW-very low birth weight; NND-neonatal death; OR-odds ratio; CI-confidence interval. Odds ratios were derived from propensity score matched analysis.
