# Supplementary material for: Associations between historical redlining and birth outcomes from 2006 through 2015 in California
Source: PLoS One. 2020 Aug 7;15(8):e0237241. doi: 10.1371/journal.pone.0237241 (PMC7413562; doi:10.1371/journal.pone.0237241)
Supplement: S4 Table — Models were adjusted for 1940s median home value, percent of employed population, percent non-white and foreign born white residents, and percent of homes reporting radio ownership. (DOCX) [file pone.0237241.s004.docx]

**S4 Table. Odds of birth outcomes by HOLC grade comparison and maternal race.**

|  |  | B vs. A |  | C vs. B |  | D vs. C |  |  |
| --- | --- | --- | --- | --- | --- | --- | --- | --- |
| PTB |  | 1.37 (1.17, 1.62) |  | 0.96 (0.95, 1.02) |  | 0.93 (0.91, 0.96) |  | Hispanic |
| LBW |  | 1.07 (0.88, 1.29) |  | 0.98 (0.94, 1.03) |  | 0.96 (0.93, 0.99) |  |  |
| SGA |  | 1.33 (1.14, 1.54) |  | 1.01 (0.97, 1.04) |  | 0.94 (0.92, 0.97) |  |  |
| PM |  | 0.44 (0.18, 1.06) |  | 1.12 (0.83, 1.51) |  | 1.09 (0.88, 1.34) |  |  |
| PTB |  | 0.97 (0.85, 1.12) |  | 0.88 (0.82, 0.94) |  | 1.07 (1.00, 1.14) |  | NH-Asian |
| LBW |  | 0.95 (0.81, 1.11) |  | 0.99 (0.91, 1.08) |  | 1.06 (0.98, 1.14) |  |  |
| SGA |  | 0.94 (0.84, 1.05) |  | 0.98 (0.93, 1.03) |  | 1.03 (0.98, 1.09) |  |  |
| PM |  | 0.86 (0.34, 2.17) |  | 0.46 (0.26, 0.82) |  | 0.97 (0.54, 1.74) |  |  |
| PTB |  | 0.98 (0.75, 1.29) |  | 1.18 (1.08, 1.29) |  | 0.97 (0.91, 1.04) |  | NH-Black |
| LBW |  | 0.98 (0.74, 1.29) |  | 1.12 (1.02, 1.22) |  | 0.94 (0.88, 1.02) |  |  |
| SGA |  | 0.93 (0.75, 1.16) |  | 0.98 (0.91, 1.05) |  | 0.95 (0.90, 1.01) |  |  |
| PM |  | 0.70 (0.13, 3.88) |  | 2.10 (1.09, 4.02) |  | 0.84 (0.55, 1.29) |  |  |
| PTB |  | 0.77 (0.54, 1.10) |  | 1.13 (0.98, 1.31) |  | 0.92 (0.80, 1.06) |  | NH-Other |
| LBW |  | 0.98 (0.64, 1.49) |  | 1.03 (0.89, 1.21) |  | 0.87 (0.85, 1.01) |  |  |
| SGA |  | 0.96 (0.68, 1.36) |  | 1.06 (0.93, 1.19) |  | 0.86 (0.77, 0.97) |  |  |
| PM |  | - |  | 0.97 (0.49, 1.91) |  | 0.53 (0.22, 1.26) |  |  |
| PTB |  | 0.89 (0.78, 1.01) |  | 1.01 (0.59, 1.71) |  | 0.96 (0.90, 1.02) |  | NH-White |
| LBW |  | 0.88 (0.76, 1.02) |  | 1.03 (0.97, 1.10) |  | 0.89 (0.82, 0.96) |  |  |
| SGA |  | 1.02 (0.91, 1.16) |  | 1.04 (0.99, 1.09) |  | 0.93 (0.87, 0.99) |  |  |
| PM |  | 0.99 (0.31, 3.10) |  | 0.91 (0.56, 1.49) |  | 1.97 (1.22, 3.18) |  |  |

Models were adjusted for 1940s median home value, percent of employed population, percent non-white and foreign born white residents, and percent of homes reporting radio ownership.
