# Supplementary material for: Associations between historical redlining and birth outcomes from 2006 through 2015 in California
Source: PLoS One. 2020 Aug 7;15(8):e0237241. doi: 10.1371/journal.pone.0237241 (PMC7413562; doi:10.1371/journal.pone.0237241)
Supplement: S5 Table — Abbreviations: LA-Los Angeles; OAK-Oakland; SF-San Francisco; PTB-preterm birth; LBW-low birth weight; SGA-small for gestational age; PM-perinatal mortality; OR-odds ratio; CI-confidence interval. (DOCX) [file pone.0237241.s005.docx]

**S5 Table. Odds ratios for birth outcomes in SF, OAK and LA from unrestricted analysis.**

|  |  |  | B vs. A |  | C vs. B |  | D vs. C |
| --- | --- | --- | --- | --- | --- | --- | --- |
|  |  |  | OR (95% CI) |  | OR (95% CI) |  | OR (95% CI) |
| All births (LA, SF, OAK) | PTB |  | 1.06 (1.00, 1.12) |  | 1.04 (1.02, 1.06) |  | 0.97 (0.95, 0.98) |
|  | LBW |  | 0.98 (0.92, 1.04) |  | 1.05 (1.02, 1.07) |  | 0.97 (0.95, 0.99) |
|  | SGA |  | 0.96 (0.91, 1.00) |  | 1.08 (1.06, 1.10) |  | 0.97 (0.95, 0.98) |
|  | PM |  | 1.32 (0.82, 2.13) |  | 1.27 (1.09, 1.49) |  | 1.15 (1.02, 1.29) |
| LA only | PTB |  | 1.06 (1.00, 1.13) |  | 1.05 (1.03, 1.07) |  | 0.98 (0.96, 0.99) |
|  | LBW |  | 0.94 (0.88, 1.01) |  | 1.05 (1.03, 1.08) |  | 0.96 (0.94, 0.98) |
|  | SGA |  | 0.95 (0.90, 1.00) |  | 1.09 (1.07, 1.11) |  | 0.97 (0.95, 0.98) |
|  | PM |  | 1.08 (0.65, 1.80) |  | 1.29 (1.09, 1.53) |  | 1.11 (0.98, 1.27) |
| SF-OAK only | PTB |  | 1.26 (1.06, 1.49) |  | 1.03 (0.96, 1.10) |  | 1.08 (1.02, 1.14) |
|  | LBW |  | 1.32 (1.09, 1.60) |  | 1.00 (0.93, 1.08) |  | 1.00 (0.95, 1.06) |
|  | SGA |  | 1.08 (0.94, 1.24) |  | 1.00 (0.95, 1.06) |  | 1.03 (0.99, 1.07) |
|  | PM |  | - |  | 1.08 (0.70, 1.68) |  | 1.14 (0.84, 1.56) |

Abbreviations: LA-Los Angeles; OAK-Oakland; SF-San Francisco; PTB-preterm birth; LBW-low birth weight; SGA-small for gestational age; PM-perinatal mortality; OR-odds ratio; CI-confidence interval
