# Supplementary material for: Associations between historical redlining and birth outcomes from 2006 through 2015 in California
Source: PLoS One. 2020 Aug 7;15(8):e0237241. doi: 10.1371/journal.pone.0237241 (PMC7413562; doi:10.1371/journal.pone.0237241)
Supplement: S6 Table — Abbreviations: N-number; %-percentage; NH-non-Hispanic. (DOCX) [file pone.0237241.s006.docx]

**S6 Table. Maternal race by HOLC grade and metropolitan area.**

|  |  | LA (N (%)) | | |  | SF-OAK (N (%)) | | |  |
| --- | --- | --- | --- | --- | --- | --- | --- | --- | --- |
|  |  | B | C | D |  | B | C | D |  |
| Hispanic |  | 33217 (44.8) | 218958 (63.3) | 77347 (73.1) |  | 2465 (10.1) | 11982 (22.8) | 13962 (37.4) | Overall |
| NH-API |  | 10314 (13.9) | 43224 (12.5) | 9198 (8.7) |  | 7557 (31.0) | 11989 (22.8) | 7717 (20.7) |  |
| NH-Black |  | 5609 (7.6) | 29196 (8.4) | 6064 (5.7) |  | 1644 (6.7) | 5676 (10.8) | 4678 (12.5) |  |
| NH-Other |  | 2361 (3.2) | 7222 (2.1) | 2322 (2.2) |  | 1645 (6.7) | 3446 (6.6) | 1851 (5.0) |  |
| NH-White |  | 22717 (30.6) | 47512 (13.7) | 10815 (10.2) |  | 11081 (45.4) | 19443 (37.0) | 9126 (24.4) |  |

Abbreviations: N-number; %-percentage; NH-non-Hispanic
