# Supplementary material for: Associations between historical redlining and birth outcomes from 2006 through 2015 in California
Source: PLoS One. 2020 Aug 7;15(8):e0237241. doi: 10.1371/journal.pone.0237241 (PMC7413562; doi:10.1371/journal.pone.0237241)
Supplement: S7 Table — Abbreviations: N-number; %-percentage; IQR-interquartile range; SD-standard deviation. (DOCX) [file pone.0237241.s007.docx]

**S7 Table. 1940s areal weighted census tract metrics by HOLC grade.**

|  |  |  | **Grade A** |  | **Grade B** |  | **Grade C** |  | **Grade D** |
| --- | --- | --- | --- | --- | --- | --- | --- | --- | --- |
| Total | N (%) |  | 83 (13.1%) |  | 203 (32.1%) |  | 239 (37.8%) |  | 108 (17.0%) |
| *Demographics* | *% Non-white (IQR)* |  | 1.4 (2.2) |  | 0.7 (1.4) |  | 0.7 (1.3) |  | 1.4 (2.7) |
| *Median home value* | *Thousands of dollars (SD)* |  | 8.1 (3.4) |  | 5.7 (2.5) |  | 4.3 (1.7) |  | 3.1 (1.3) |
| *Median rent* | *Dollars (SD)* |  | 40.7 (25.2) |  | 35.5 (12.0) |  | 30.0 (9.0) |  | 23.7 (7.3) |
| *Population per dwelling* | *Persons per unit (IQR)* |  | 3.3 (0.4) |  | 3.2 (0.3) |  | 3.1 (0.4) |  | 3.2 (0.4) |
| *Proportion of homes needing repairs* | *No repairs (SD)* |  | 90.7 (7.1) |  | 90.1 (5.8) |  | 88.4 (7.5) |  | 86.0 (9.6) |
|  | *Major repairs (SD)* |  | 2.5 (4.2) |  | 3.6 (4.0) |  | 4.7 (6.4) |  | 8.1 (9.1) |
|  | *Not reported (SD)* |  | 6.8 (6.2) |  | 6.3 (4.5) |  | 6.9 (5.4) |  | 5.9 (5.0) |

Abbreviations: N-number; %-percentage; IQR-interquartile range; SD-standard deviation.
